# Downregulation of snoRNA SNORA52 and Its Clinical Significance in Hepatocellular Carcinoma

**DOI:** 10.1155/2021/7020637

**Published:** 2021-06-03

**Authors:** Yuan Ding, Zhongquan Sun, Sitong Zhang, Yanjie Li, Xin Han, Qianhui Xu, Liuzhi Zhou, Hao Xu, Yang Bai, Chang Xu, Hao Ding, Yao Ge, Weilin Wang

**Affiliations:** ^1^Department of Hepatobiliary and Pancreatic Surgery, The Second Affiliated Hospital, Zhejiang University School of Medicine, Hangzhou, Zhejiang 310009, China; ^2^Key Laboratory of Precision Diagnosis and Treatment for Hepatobiliary and Pancreatic Tumor of Zhejiang Province, Hangzhou, Zhejiang 310009, China; ^3^Research Center of Diagnosis and Treatment Technology for Hepatocellular Carcinoma of Zhejiang Province, Hangzhou, Zhejiang 310009, China; ^4^Clinical Medicine Innovation Center of Precision Diagnosis and Treatment for Hepatobiliary and Pancreatic Disease of Zhejiang University, Hangzhou, Zhejiang 310009, China; ^5^Clinical Research Center of Hepatobiliary and Pancreatic Diseases of Zhejiang Province, Hangzhou, Zhejiang 310009, China

## Abstract

Hepatocellular carcinoma (HCC) is one of the most common and aggressive tumors in the world while the accuracy of the present tests for detecting HCC is poor. A novel diagnostic and prognostic biomarker for HCC is urgently needed. Overwhelming evidence has demonstrated the regulatory roles of small nucleolar RNA (snoRNA) in carcinogenesis. This study is aimed at analyzing the expression of a snoRNA, SNORA52, in HCC and exploring the correlation between its expression and various clinical characteristics of HCC patients. By using quantitative real-time PCR, we found that SNORA52 was downregulated in HCC cell lines (*P* < 0.05) and HCC tissues (*P* < 0.001). Correlation analysis showed that the expression of SNORA52 was obviously associated with tumor size (*P* = 0.011), lesion number (*P* = 0.007), capsular invasion (*P* = 0.011), tumor differentiation degree (*P* = 0.046), and TNM stage (*P* = 0.004). The disease-free survival (DFS) and overall survival (OS) analysis showed that patients with lower SNORA52 expression had a worse prognosis (*P* < 0.001). Univariate and multivariate Cox regression analysis showed that SNORA52 expression was a completely independent prognostic factor to predict DFS (*P* = 0.009) and OS (*P* = 0.012) of HCC patients. Overall, our findings showed SNORA52 expression levels were downregulated in HCC tissues and correlated with multiple clinical variables, and SNORA52 was an independent prognostic factor for HCC patients, which suggested that SNORA52 could function as a potential diagnostic and prognostic biomarker for HCC patients.

## 1. Introduction

Hepatocellular carcinoma (HCC) is the main type of liver cancer [1] as well as the seventh most common cancer in the world [2]. As the tumor usually progresses with a concealed onset, most HCC are diagnosed at its advanced stage, which commonly indicates limited treatments and poor prognosis for patients [3]. Even after R0 surgical resection, HCC recurrence remains up to 50%-70% in postoperative five years [4]. Among that, less than 50% of those with significant portal hypertension and abnormal bilirubin could survive five years [5], which constitutes the reason why liver cancer is still the second most lethal cancer in the world, causing more than 830,000 deaths [2]. Based on this, accurate early diagnosis and long-term prognostic prediction held an important position in the clinical management of HCC. For a long time, *α*-fetoprotein (AFP) has been the most common tumor marker for HCC. However, the accuracy of the AFP test is disappointing, showing only about 60% of sensitivity and 80% of specificity even in its most efficient cutoff (10–20 ng/mL). Other tumor markers, including protein induced by vitamin K absence or antagonist-II (PIVKA-II), have not provided better accuracy neither [6, 7]. Therefore, a novel diagnostic and prognostic biomarker for HCC is urgently needed.

Recently, overwhelming evidence has demonstrated the regulatory roles of different classes of noncoding RNAs (ncRNAs) in a carcinogenesis process [8]. Considered one of the best-characterized classes of ncRNAs, small nucleolar RNAs (snoRNAs) typically make functions in posttranscriptional modification of ribosomal and spliceosomal RNAs [9]. However, unbiased screens for cancer genes indicated some unexpected roles for snoRNAs in cancer. Emerging evidences indicated that snoRNAs were able to directly affect HCC development by regulating several pathways, such as colony formation and cellular apoptosis [10, 11]. For example, Xu et al. demonstrated that SNORD113-1 could inhibit the phosphorylation of extracellular signal-regulated kinase 1/2 (ERK1/2) and then functionally suppress HCC growth. Meanwhile, downregulated expression of SNORD113-1 was significantly associated with poor long-term survival in HCC patients [12]. Besides, SNORD126 was proved to be overexpressed in HCC and promote HCC growth via activating the PI3K-AKT pathway [13].

Small nucleolar RNA SNORA52, a novel H/ACA box snoRNA, was newly found to perform latent regulatory functions in tumor progression. Schulten et al. revealed that dysregulation of SNORA52 could indicate severe metastasis and invasion characteristics in breast as well as brain malignant tumors through comprehensive molecular biomarker identification [14]. What is more, by reference to the biofunctional predictive analysis, SNORA52 was found to be related to cell cycle, DNA replication, and repairing. However, the research of potential relationship between SNORA52 and HCC remains blank.

In the present study, we analyzed the abnormal SNORA52 expression in HCC and explored the correlation between SNORA52 expression and various clinical characteristics of HCC patients.

## 2. Materials and Methods

### 2.1. Patients and Specimens

Surgical HCC and paired adjacent liver specimens were obtained from 145 HCC patients (from 26 to 82 years old) who had hepatectomy from January 2013 to August 2014. None of the patients had received preoperative chemotherapy or radiotherapy treatment before hepatectomy. All patients were diagnosed with HCC by reference of histological examination.

The perioperative data of patients were collected from a hospital medical database system, including age, gender, hepatitis B virus infection, liver cirrhosis, AFP, tumor diameter, and TNM stages. Valid survival data was obtained over a period of 60 months.

### 2.2. Cell Culture

The human normal hepatocyte cell line QSG-7701 and several human HCC cell lines (SK-HEP-1 and Huh-7) were cultured in DMEM (Gibco, USA) containing heat-inactivated 10% fetal bovine serum (FBS, Gibco, USA), 100 mg/mL streptomycin, and 100 U/mL penicillin in a humidified atmosphere of 5% CO_2_ at 37°C.

### 2.3. RNA Extraction and Reverse Transcription

Total RNA from cells and tissues were extracted using Trizol reagent (Invitrogen, Carlsbad, CA, USA). The concentration and purity of RNA were quantified by Nanodrop 2000 spectrophotometer (Thermo Fisher Scientific Inc., Waltham, MA, 93 USA). Then, total RNA was reverse transcribed to cDNA using HiScript II Reverse Transcriptase SuperMix with gDNA wiper (Vazyme, Nanjing, China) according to the manufacturer's instructions.

### 2.4. Real-Time PCR Analysis

According to the manufacturer's instructions of Fast Start Universal SYBR Green Master ROX (Roche, Basel, Switzerland), the expressions of SNORA52 were measured through quantitative real-time polymerase chain reaction (RT-PCR). Ct values of the target SNORA52 were normalized in relation to GAPDH. The comparative Ct method formula 2^-*ΔΔ*Ct^ was used to calculate the relative gene expression. All samples were tested in triplicates. The primers were designed based on SNORA52 gene sequence obtained from the gene database of NCBI. The primer sequences were as follows (5′-3′): SNORA52—GTCCATCCTAATCCCTGCCG (forward), CTAGAAGTGCCCATGACGTGAG (reverse); GAPDH—CAGGAGGCATTGCTGATGAT (forward), GAAGGCTGGGGCTCATTT (reverse).

### 2.5. Statistical Analysis

All statistical analysis was carried out using the SPSS19.0 (SPSS Inc., Chicago, IL, USA). All *P* values shown were two-sided, and *P* < 0.05 was considered statistically significant. The quantitative variables were evaluated by Student's *t*-test, while the chi-square test and Fisher's exact test were applied to analyze qualitative and categorical data. Overall survival curves were plotted with the use of the Kaplan-Meier method. Overall, the association of SNORA52 expression and prognosis survival were plotted with the use of univariate analysis and multivariate Cox regression model.

## 3. Results and Discussion

### 3.1. SNORA52 Expression Level Was Significantly Lower in Hepatocarcinoma Cell Lines

**Figure 1:**
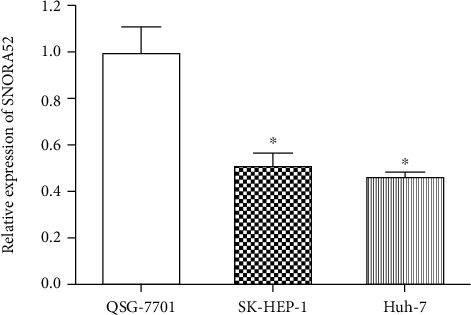
Analysis of SNORA52 expression in HCC and normal liver cell lines. Compared to normal liver cell line QSG-7701, SNORA52 was notably downexpressed in several HCC cell lines: SK-HEP-1 (*P* = 0.013) and Hep 3B (*P* = 0.032). ^∗^*P* < 0.05.

Comparing to human hepatocytes QSG-7701, significantly lower expression of SNORA52 was detected in HCC cell lines of both SK-HEP-1 and Huh-7 (*P* < 0.05, Figure 1), which suggested that SNORA52 might play a tumor suppressing role in HCC.

### 3.2. SNORA52 Was Downregulated in HCC Tissues

**Figure 2:**
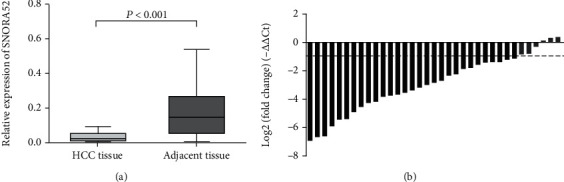
Relative expression of SNORA52 in clinical HCC and adjacent normal tissues. (a) SNORA52 expression was significantly lower in HCC tissues than in adjacent normal tissues (*P* < 0.001). (b) Waterfall plot showed the fold change of SNORA52 expression. Relative expression = 2^−ΔΔCt^, −ΔΔCt = (CtGAPDH–CtSNORA52) of HCC tissues–(CtGAPDH–CtSNORA52) of adjacent liver tissue.

To further verify the discovery of SNORA52 downregulation in HCC, we performed the comparison of SNORA52 expression in pairs of HCC tissues and adjacent liver tissues. Consistent with cellular experiment above, we found that SNORA52 expression was significantly lower in HCC tissues than in adjacent liver tissues (*P* < 0.001, Figure 2).

### 3.3. Correlation between SNORA52 Expression and Clinical Variables

According to individual SNORA52 expression, all 145 HCC patients were classified into the low and high expression subgroups. As shown in Table 1, two SNORA52 subgroups were qualitatively compared in aspects of several clinical characteristics. Correlation analysis showed that SNORA52 expression levels in HCC tissues were significantly associated with tumor size (<5 cm *vs.* ≥5 cm, *P* = 0.011), lesion number (single *vs.* multiple, *P* = 0.007), capsular invasion (absent *vs.* present, *P* = 0.011), tumor differentiation degree (high/moderate *vs.* low, *P* = 0.046), and TNM stage (I∼II *vs.* III∼IV, *P* = 0.004). These results indicated that SNORA52 expression was remarkably associated with the malignancy of HCC lesions in patients.

### 3.4. Association between SNORA52 Levels and Disease-Free Survival

**Figure 3:**
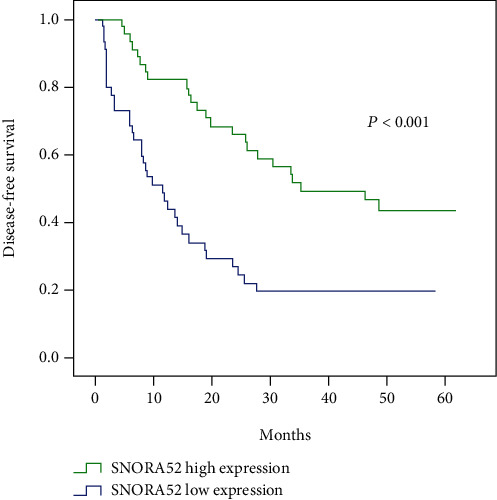
Cumulative disease-free survival curves of patients in high and low SNORA52 expression groups.

In order to explore the prognostic value of SNORA52 in HCC, we assessed the relationship between SNORA52 expression and disease-free survival (DFS) using the Kaplan-Meier method. Obviously, compared with the high SNORA52 expression group, the patients in the low SNORA52 expression group were inclined to present with higher tumor recurrence rate and shorter disease-free survival time (*P* < 0.001, Figure 3). This significant difference indicates that SNORA52 expression had potential capability to predict the disease-free survival of HCC patients.

What is more, the following univariate and multivariate Cox regression analyses were conducted to assess the independent prognostic indicators for HCC patients (Table 2). Firstly, univariate analysis showed that tumor size, lesion number, capsular invasion, vessel carcinoma embolus, TNM stage, and SNORA52 expression were strongly associated with the DFS of HCC patients. Then, multivariate analysis was performed within the above characteristics. Ultimately, it confirmed that SNORA52 expression (*P* = 0.009), tumor number (*P* = 0.007), and vessel carcinoma embolus (*P* = 0.014) were the completely independent prognostic factors to predict DFS of HCC patients.

### 3.5. Association between SNORA52 Levels and Overall Survival

**Figure 4:**
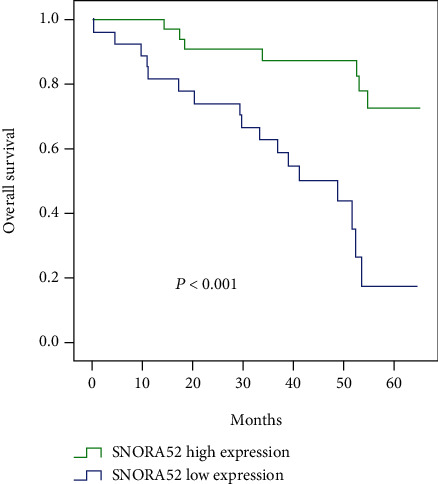
Cumulative overall survival curves of patients in the high and low SNORA52 expression groups.

Similarly, we assessed the relationship between the SNORA52 expression and overall survival (OS) under Kaplan-Meier method. Patients from the low SNORA52 expression group were inclined to present with more severe overall survival than patients with high SNORA52 expression (*P* < 0.001, Figure 4), indicating that there was some potential correlation between SNORA52 expression and HCC overall survival.

As shown in Table 3, univariate Cox regression analysis showed that tumor size, tumor number, capsular invasion, vessel carcinoma embolus, TNM stage, and SNORA52 expression were significantly associated with overall survival of HCC patients. Moreover, following multivariate analysis confirmed that SNORA52 expression (*P* = 0.012), tumor size (*P* = 0.001), tumor number (*P* = 0.001), and vessel carcinoma embolus (*P* = 0.002) all could function as independent indicators in predicting overall survival of HCC patients.

## 4. Discussion

As the second most lethal tumor in the world [15], HCC commonly progress without apparent symptom and almost 60% of HCC patients are firstly diagnosed in intermediate or advanced stages [16]. Thus, accurately detecting HCC at an early stage is helpful for improving overall survival [17]. Clinically, biomarkers are more appropriately used for a surveillance test while ultrasonography is not widely available owing to limited resources [18]. However, even as the most commonly used biomarker, AFP maintains a controversial clinical significance with the dissatisfactory sensitivity of 40–60% [19]. Besides, postoperative recurrence and prognosis prediction are also key obstacles in HCC treatment lacking relevant indicators [20]. Based on these, a novel biomarker is eagerly needed for the clinical treatment of HCC.

Along with more and more mechanisms of HCC tumorigenesis being revealed, newly discovered biomarkers are evaluated for diagnosis, assessment, or treatment of HCC patients [21, 22]. In recent years, mounting evidence has indicated the direct relationship between ncRNAs' dysfunction and tumor oncogenesis as well as the function of ncRNAs as assessment indicators for tumor progression [23]. Constituting almost 60% of transcripts in human cells, ncRNAs are not directly translated into proteins but exert regulatory functions in various cellular physiological as well as pathological processes [24].

Among that, snoRNA, as one kind of ncRNAs within 200 nucleotides length, is commonly found located in the nucleolus and make regulatory functions in the process of posttranscriptional modification [25]. Following the advances in the field of tumor regulation, it has proved that the dysfunction of specific snoRNA could directly induce and promote the development of various tumors [26], including HCC. Actually, Xu's team is the first to illustrate the relationship between snoRNA and HCC, which indicated that snoRNA SNORD113-1 in HCC could inactivate the intracellular phosphorylation of ERK1/2 and SMAD2/3, presenting its tumor-suppressing function [12]. On the contrary, the overexpressed SNORD126 in HCC was proved to function as the tumor-promoting snoRNA through increasing fibroblast growth factor receptor 2 (FGFR2) expression and then activating the PI3K-AKT pathway [13]. What is more, according to a recent study by McMahon et al., HCC patients with low SNORA24 expression commonly tended to exhibit poor long-term survival [27]. Apparently, the dysregulation of some specific snoRNAs possesses direct influence on both HCC progression and a patient's condition.

In the present study, we for the first time investigated the potential association between SNORA52 expression and HCC. With validation of both cell lines and clinical specimens, we verified that SNORA52 was notably downregulated in HCC. Furthermore, it was found that the SNORA52 expression was significantly associated with HCC malignancy classification, including tumor size, lesion number, capsular invasion, tumor differentiation, and TNM stage. In addition, combining Kaplan–Meier analysis with a Cox regression model, we certified that SNORA52 expression had independent predictive capability in aspects of postoperative tumor recurrence and long-term survival. We also notice that Yang et al. did not include SNORA52 as an analytic target in their genomic analysis of snoRNAs basing on online database [28]. Given that the snoRNA expression profiles may vary from one race, nationality, database, or analytic method to another, we are prone to rely on the data acquired from our tangible specimens. Summarizing the above results, we supposed that SNORA52 played a suppressing role in the occurrence and progression of HCC.

Typically, snoRNAs were divided into C/D box and H/ACA box subtypes for their different modifications in rRNAs [29]. Among that, the H/ACA box snoRNAs, which SNORA52 belongs to, commonly modulate rRNA-related functions by pseudouridylation, while abnormal rRNA pseudouridylation can directly promote oncogenesis [30, 31]. By reference to the study of Bellodi et al. for example, the deficiency of one pseudouridine synthase dyskerin could contribute to spontaneous pituitary tumorigenesis by blocking the transcription of tumor suppressor p27 [32]. Moreover, mutations in H/ACA box RNPs, each consisting of one unique snoRNA and 4 common core proteins, accounted for the oncogenesis of prostatic carcinoma and other cancers [33]. What is more, the downregulation of NHP2, derived from one H/ACA box RNP catalyzing rRNA pseudouridylation, could result in cyst formation [34]. Taking a concrete example, Liu et al. found SNORA23 inhibited HCC progress by impairing methylation of 28S rRNA, which regulates ribosome biogenesis [35]. Based on these, we assume that the downregulated SNORA52 in HCC could cause dysfunction of some following tumor suppressor rRNAs or ribosomes, and finally, it promoted HCC oncogenesis and development. However, to validate the exact mechanisms of SNORA52 in HCC, more related researches will be needed in the future.

## 5. Conclusions

In summary, we for the first time found that SNORA52 expression levels were downregulated in HCC tissues and notably correlated with multiple clinical variables. Furthermore, the survival analysis indicated that SNORA52 was an independent prognostic factor for HCC patients. These results suggested that SNORA52 could function as a potential diagnostic and prognostic biomarker for HCC patients.

## Figures and Tables

**Table 1:** Correlation of SNORA52 and clinical characteristics in HCC patients.

Characteristics	Total	SNORA52 expression		*P* value
		Low (*n* = 72)	High (*n* = 73)	
Age (ys)				0.607
<60	92	44 (0.611)	48 (0.658)	
≥60	53	28 (0.389)	25 (0.342)	
Gender				0.138
Female	19	6 (0.083)	13 (0.178)	
Male	126	66 (0.917)	60 (0.822)	
Hepatitis B				0.810
Positive	125	63 (0.875)	62 (0.849)	
Negative	20	9 (0.125)	11 (0.151)	
Cirrhosis				1.000
Present	89	44 (0.611)	45 (0.616)	
Absent	56	28 (0.389)	28 (0.384)	
AFP (ng/l)				0.230
<400	92	42 (0.583)	50 (0.685)	
≥400	53	30 (0.417)	23 (0.315)	
Tumor diameter				0.011
<5 cm	43	14 (0.194)	29 (0.397)	
≥5 cm	102	58 (0.806)	44 (0.603)	
Multiple lesions				0.007
Absent	24	18 (0.250)	6 (0.082)	
Present	121	54 (0.750)	67 (0.918)	
Vessel carcinoma embolus				0.127
Absent	109	50 (0.694)	59 (0.808)	
Present	36	22 (0.306)	14 (0.192)	
Microvascular invasion				1.000
Absent	136	68 (0.944)	68 (0.932)	
Present	9	4 (0.056)	5 (0.068)	
Capsular invasion				0.011
Absent	88	36 (0.500)	52 (0.712)	
Present	57	36 (0.500)	21 (0.288)	
Differentiation				0.046
Low	76	44 (0.377)	32 (0.438)	
High/moderate	69	28 (0.389)	41 (0.562)	
TNM stage				0.004
I~II	101	42 (0.583)	59 (0.808)	
III~IV	44	30 (0.417)	14 (0.192)	

AFP: *α*-fetoprotein; TNM: tumor-node-metastasis; ^∗^*P* < 0.05. Values are presented as the mean ± standard deviation or *n* (%).

**Table 2:** Univariate and multivariate analysis of disease-free survival in HCC patients.

Clinicopathologic parameters	Univariate analysis		Multivariate analysis	
	HR (95% CI)	*P*	HR (95% CI)	*P*
Age (<60 vs. ≥60)	0.637 (0.371-1.093)	0.102		
Gender (female vs. male)	1.506 (0.647-3.506)	0.342		
Hepatitis B (negative vs. positive)	1.729 (0.785-3.810)	0.174		
AFP (<400 vs. ≥400)	1.355 (0.808-2.273)	0.249		
Cirrhosis (present vs. absent)	1.302 (0.764-2.221)	0.332		
Microvascular invasion (present vs. absent)	0.931 (0.337-2.573)	0.891		
Tumor differentiation (low vs. high/moderate)	1.018 (0.611-1.698)	0.944		
Capsular invasion (present vs. absent)	1.575 (0.939-2.644)	0.085	1.272 (0.753-2.147)	0.369
TNM stage (I~II vs. III~IV)	2.008 (1.190-3.386)	0.009	1.094 (0.550-2.175)	0.798
Tumor diameter (<5 cm vs. ≥5 cm)	1.875 (1.010-3.482)	0.046	1.861 (0.969-3.571)	0.062
Multiple lesions (present vs. absent)	2.481 (1.392-4.419)	0.002	2.229 (1.245-3.993)	0.007∗
Vessel carcinoma embolus (present vs. absent)	2.250 (1.260-4.019)	0.006	2.195 (1.169-4.120)	0.014∗
SNORA52 expression (low vs. high)	0.374 (0.220-0.634)	<0.001	0.481 (0.277-0.836)	0.009∗

AFP: *α*-fetoprotein; TNM: tumor-node-metastasis; ^∗^*P* < 0.05 was considered to be statistically significant.

**Table 3:** Univariate and multivariate analysis of overall survival in HCC patients.

Clinicopathologic parameters	Univariate analysis		Multivariate analysis	
	HR (95% CI)	*P*	HR (95% CI)	*P*
Age (<60 vs. ≥60)	1.153 (0.572-2.322)	0.691		
Gender (female vs. male)	0.896 (0.344-2.331)	0.822		
Hepatitis B (negative vs. positive)	1.056 (0.406-2.746)	0.911		
AFP (<400 vs. ≥400)	1.614 (0.805-3.235)	0.177		
Cirrhosis (present vs. absent)	0.918 (0.453-1.861)	0.812		
Microvascular invasion (present vs. absent)	1.255 (0.382-4.126)	0.708		
Tumor differentiation (low vs. high/moderate)	0.724 (0.357-1.469)	0.371		
Capsular invasion (present vs. absent)	2.143 (1.070-4.293)	0.032	1.132 (0.530-2.419)	0.748
TNM stage (I~II vs. III~IV)	2.712 (1.348-5.455)	0.005	0.803 (0.299-2.157)	0.663
Tumor diameter (<5 cm vs. ≥5 cm)	5.113 (1.553-16.834)	0.007	9.292 (2.457-35.137)	0.001
Multiple lesions (present vs. absent)	3.607 (1.725-7.542)	0.001	3.548 (1.654-7.612)	0.001
Vessel carcinoma embolus (present vs. absent)	2.052 (0.919-4.582)	0.079	4.519 (1.737-11.759)	0.002
SNORA52 expression (low vs. high)	0.274 (0.127-0.594)	0.001	0.359 (0.161-0.802)	0.012

AFP: *α*-fetoprotein; TNM: tumor-node-metastasis; ^∗^*P* < 0.05 was considered to be statistically significant.

## Data Availability

All data generated or analyzed during this study are included in this published article.
