# Correction to “Downregulation of snoRNA SNORA52 and Its Clinical Significance in Hepatocellular Carcinoma”

**DOI:** 10.1155/bmri/9768387

**Published:** 2025-09-30

Y. Ding, Z. Sun, S. Zhang, et al., “Downregulation of snoRNA SNORA52 and Its Clinical Significance in Hepatocellular Carcinoma,” *BioMed Research International* 2021 (2021): 7020637, https://doi.org/10.1155/2021/7020637.

In the article, there is an error in Figure 4. The authors explained that Figure 4 was generated mistakenly using a wrong dataset but when they generated the correct graph, the same trend was observed. As the same trend was observed, the authors confirm that these errors do not affect the conclusions of the article and this has been confirmed by the journal’s Editorial Board. The correct Figure 4 is shown below:

**Figure 4:**
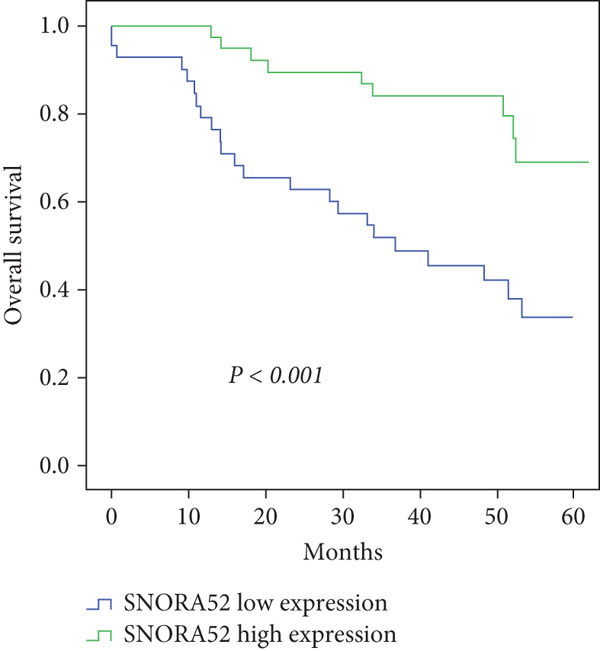
Cumulative overall survival curves of patients in the high and low SNORA52 expression groups.

We apologize for this error.

